# Simulating photon scattering effects in structurally detailed ventricular models using a Monte Carlo approach

**DOI:** 10.3389/fphys.2014.00338

**Published:** 2014-09-09

**Authors:** Martin J. Bishop, Gernot Plank

**Affiliations:** ^1^Division of Imaging Sciences & Biomedical Engineering, Department of Biomedical Engineering, King's College LondonLondon, UK; ^2^Institute of Biophysics, Medical University of GrazGraz, Austria; ^3^Oxford eResearch Centre, University of OxfordOxford, UK

**Keywords:** optical mapping, photon scattering, cardiac modeling, Monte Carlo, bidomain

## Abstract

Light scattering during optical imaging of electrical activation within the heart is known to significantly distort the optically-recorded action potential (AP) upstroke, as well as affecting the magnitude of the measured response of ventricular tissue to strong electric shocks. Modeling approaches based on the photon diffusion equation have recently been instrumental in quantifying and helping to understand the origin of the resulting distortion. However, they are unable to faithfully represent regions of non-scattering media, such as small cavities within the myocardium which are filled with perfusate during experiments. Stochastic Monte Carlo (MC) approaches allow simulation and tracking of individual photon “packets” as they propagate through tissue with differing scattering properties. Here, we present a novel application of the MC method of photon scattering simulation, applied for the first time to the simulation of cardiac optical mapping signals within unstructured, tetrahedral, finite element computational ventricular models. The method faithfully allows simulation of optical signals over highly-detailed, anatomically-complex MR-based models, including representations of fine-scale anatomy and intramural cavities. We show that optical action potential upstroke is prolonged close to large subepicardial vessels than further away from vessels, at times having a distinct “humped” morphology. Furthermore, we uncover a novel mechanism by which photon scattering effects around vessels cavities interact with “virtual-electrode” regions of strong de-/hyper-polarized tissue surrounding cavities during shocks, significantly reducing the apparent optically-measured epicardial polarization. We therefore demonstrate the importance of this novel optical mapping simulation approach along with highly anatomically-detailed models to fully investigate electrophysiological phenomena driven by fine-scale structural heterogeneity.

## 1. Introduction

Cardiac optical mapping provides high-resolution spatiotemporal recordings of electrophysiological activity from the surface of myocardial tissue (Efimov et al., 2004). The method utilizes specialized membrane-bound fluorescent dyes, which, upon illumination at the correct wavelength, transduce local changes in transmembrane potential as changes in fluorescent emission, which can be recorded by optical detectors. However, light is known to be highly scattering and relatively weakly absorbing in cardiac tissue at both the excitation and emission wavelengths of the voltage-sensitive fluorescent dyes used. Illuminating light penetrates relatively deeply (a few millimeters) into the tissue, with the subsequent emitted fluorescence also scattering and escaping the surface to be detected. Consequently, the measured fluorescent signals are thought to contain information regarding the electrophysiological state of, not just the tissue surface, but of tissue within a three-dimensional “scattering volume” underneath the surface recording site. The resulting effect of averaging electrical states within a subsurface volume is known to give rise to distortion in recorded fluorescent signals relative to measurements obtained with micro-electrode recordings and as well as those predicted by computational models. Such distortion effects include the prolongation of the optically-recorded action potential upstroke duration (Girouard et al., 1996; Gray, 1999; Hyatt et al., 2003, 2005; Bishop et al., 2006a), the modulation of surface-recorded shock-end polarization levels following strong extracellular shocks (Janks and Roth, 2002; Bishop et al., 2006b, 2007) as well as the appearance of “dual-humped” action potentials from recording sites above intramural reentry (Efimov et al., 1999, 2000; Bray and Wikswo, 2003; Bishop et al., 2007).

In recent decades, computational models of cardiac electrophysiological dynamics have been instrumental in enriching our understanding of a variety of physiological and pathological cardiac phenomena. Optical mapping measurements provide a key component in fully utilizing the predictions made from such models, facilitating an important form of experimental comparison and model validation. Using models directly alongside experimental measurements often also allows for a more detailed mechanistic understanding of particular experimental findings. However, the presence of distortion effects due to photon scattering in optical mapping has the potential to render such a direct comparison with models problematic, limiting the use of optical recordings to validate simulations of electrical activity and compromising the interpretation of experimental mapping data.

To combat these issues, combined electrophysiological and optical simulation models have been developed to synthesize the fluorescent signals recorded in optical mapping experiments. The use of such combined models have provided important post-processing tools which can be applied to “raw” electrophysiological simulation data derived from standard mono-/bidomain simulations. As they intrinsically simulate the presence of the distortion artifact, they consequently provide a much closer comparison with experimental optical mapping recordings, improving the validation procedure of simulation results. Furthermore, their ability to simulate each stage of the optical mapping process facilitates a much more complete mechanistic understanding of the origin of fluorescent signal distortion, suggesting ways in which novel interpretation of the recorded signal, guided by the models, may provide invaluable information regarding intramural electrophysiological activity (Hyatt et al., 2003, 2005; Bishop et al., 2007; Walton et al., 2010).

By far the most common models used to simulate the fluorescent optical signals have been continuum models, based on the photon diffusion equation, valid as light is relatively highly scattering in biological tissue at the wavelengths used. Both analytical (Hyatt et al., 2003) and numerical (finite element) (Bishop et al., 2006a) solution methods for the photon diffusion equation have been successfully used; the former being largely restricted to modeling regular (plane/slab) geometries whilst the latter has been able to simulate fluorescent signals from anatomically-realistic ventricular models. However, despite providing important insight into the three-dimensional interaction between light scattering and the complex electrical activity patterns that underlie paced rhythms (Hyatt et al., 2005; Bishop et al., 2006a) as well as following strong electrical shocks and during episodes of arrhythmia (Bishop et al., 2007, 2011a), the geometrical models used in these studies have all represented the myocardial wall as solid, compact myocardium.

In recent years, there has been an advent of the construction of highly-detailed ventricular models derived from high-resolution (up to 25 μm) MR data (Plank et al., 2009; Bishop et al., 2010b). These models contain a wealth of fine-scale anatomical features such as the coronary vasculature, intramural extracellular cleft spaces in addition to endocardial structures such as papillary muscles and trabeculations, and have been used to demonstrate the potential importance of these features in mechanisms of arrhythmia and electrotherapy (Bishop et al., 2010b,a, 2012; Rantner et al., 2013). During a typical optical mapping experiment, the presence of intramural cavities (vessels, clefts) would become filled with transparent saline solution. These cavities thus represent regions of non-scattering optical media in which photon diffusion does not hold, and thus the photon diffusion equation can not be applied, preventing the use of these latest high-resolution anatomical models along with the previous optical simulation methods. The increasing use of such detailed anatomical models in computational cardiac electrophysiology thus hastens the need to develop an optical simulation tool capable of simulating light transport through both scattering and non-scattering media alike. Such a tool will be able to validate the predictions made by this new generation of models and provide further insight into experimental measurements which aim to probe the mechanisms of fine-scaled anatomy in various aspects of cardiac function.

The above requirements suggest the use of a discrete stochastic modeling approach, such as the Monte Carlo (MC) method, which is able to simulate the movement of photons throughout a medium, regardless of its optical properties. The MC method models the propagation of individual photons, or packets of photons, simulating individual scattering, absorption, reflection, and refraction processes. MC methods have been used extensively with much success in a wide variety of applications to biomedical optics phenomena, being the “Gold Standard” to which other methods (such as the photon diffusion method) are compared against due to the accuracy of their predictions, combined with the wealth of information which goes into, and which it is possible to extract from, each simulation (Wang et al., 1995; Okada et al., 1996; Jacques, 1998). As each photon interaction event occurs stochastically, each photon trajectory is unique and thus large numbers of photons need to be simulated to obtain good statistics, leading to high computational expense (Arridge, 1993; Wang et al., 1995).

Compared to photon diffusion approaches, MC methods have been relatively sparsely applied to the simulation of optical mapping signals. As individual photon trajectories are simulated, the explicit origin of each photon recorded by the optical detector is known. MC methods have thus been largely used to provide information regarding the spatial distribution of tissue contributing to the recorded optical mapping signal (i.e., the scattering volume) (Ding et al., 2001) and how this might be influenced by different illumination strategies (Ramshesh and Knisley, 2003), pixel sizes (Bishop et al., 2009) and optical detection setups (Bishop et al., 2009). Such studies have also been exclusively restricted to highly simplified geometric models, usually representing regular slabs of (solid) cardiac tissue, due to the requirement of very rapidly and efficiently computing the photon packet's location within the tissue; trivial in regular, structured grids. However, in unstructured meshes, such as those necessarily used to construct the latest anatomically-detailed models, tracking a packet's position, and in addition, checking for potential boundary interactions, in a highly-optimized computational manner becomes a significant challenge. Recently, MC models of photon propagation within unstructured tetrahedral meshes have been proposed, where the interaction between photon packet and triangular tetrahedral element face is computed recursively and rapidly (Shen and Wang, 2010), and has been applied to model light propagation within a whole body mouse mesh, including organs.

The goal of this study is to present a novel application of the MC method of photon scattering simulation within unstructured meshes, proposed by Shen and Wang (2010), applied for the first time to the simulation of cardiac voltage-sensitive fluorescent optical mapping signals. The developed approach is used to understand the origin of recorded fluorescent signals within highly anatomically-detailed ventricular models, and to specifically understand how the presence of large blood vessel cavities close to the epicardial recording surface may significantly distort the recorded optical signal during pacing and following the application of strong extracellular shocks.

## 2. Methods

### 2.1. Geometrical finite element computational models of cardiac tissue

High-resolution, unstructured tetrahedral finite element meshes representing cardiac left-ventricular (LV) wedge preparations were used throughout to simulate optical mapping signal synthesis. The models included three geometrically-simplistic cuboid models (both with and without the presence of a blood vessel) in addition to an anatomically-detailed model derived from high-resolution (25 μm) rabbit MR-data (Bishop et al., 2010a,b).

**Figure 1:**
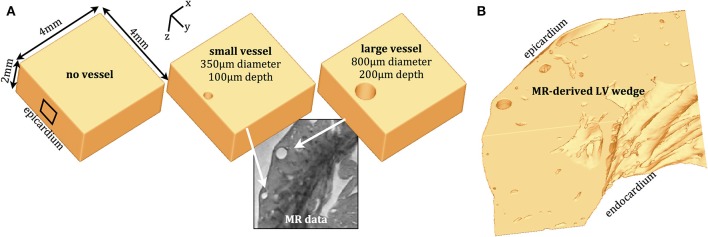
**Computational finite element models. (A)** Simple, cuboid models representing myocardial wedge preparations with no vessel (left) and with representations of small (center) and large (right) sub-epicardial intramural blood vessels, informed from high-resolution rabbit MR data (Bishop et al., 2010b). **(B)** High-resolution MR-derived LV wedge preparation (Bishop et al., 2010a).

The first simple cuboid model represented compact myocardial tissue with no cavities, of dimension 4 × 4 × 2 mm in the *x*-, *y*-, and *z*-directions, respectively. The other two cuboid models, of the same overall dimension, contained smooth cylindrical cavities of diameter 350 and 800 μm representing a blood vessel passing through the intramural ventricular wall in the global apex-base direction at respective depths of 100 and 200 μm beneath the epicardial surface (to cavity edge). Figure 1A depicts the geometrically-simple models in addition to an example MR image (Bishop et al., 2010b) of vessels used to guide the choice of cavity size and depth. The anatomically-detailed MR-derived model (Bishop et al., 2010a) was based-on a left ventricular (LV) wedge preparation of height 6 mm in the apex-base direction and represented approximately one quarter of the LV free wall, as shown in Figure 1B. The high level of anatomical detail present in the MR-images was successfully carried-over to the model, representing an intricate amount of details regarding fine-scale intramural structures such as blood vessels and extracellular clefts.

The meshing software Tarantula (CAE Solutions, Austria) was used to create the meshes directly from segmented binary voxel image stacks. For geometrically-simple models, binary image stacks were manually created in Matlab. In the case of the MR-derived model, processing, and segmentation of the MR data was performed to generate the binary mask, as described in detail in Bishop et al. (2010a,b). Meshes had a mean element discretization of approximately 50 μm within cardiac tissue.

Transversely-rotational fiber orientation was assigned to the models, rotating ± 60° between epi- and endocardial surfaces. A previously described algorithm based-on a Laplace-Dirichlet approach (Bishop et al., 2010a) was used for assigning the smooth negotiation of cardiac fibers around intramural cavities, informed from histology (Gibb et al., 2009). The electrically-insulating effects of the connective tissue surrounding blood vessel walls was represented by assigning tagged elements around vessel cavities in the meshes with reduced electrical conductivity values derived directly from experiment (Bishop et al., 2010a).

In addition to representing the myocardial tissue, the meshes also contained an unstructured finite element representation of the perfusing bath, including the bath contained within all intramural cavities (blood vessels and extracellular cleft spaces) and that surrounding the tissue on all sides, as would be the case for a perfused optical mapping preparation. For the simple cuboid models, a surrounding bath of width 100 μm (approximately two element widths) was modeled. For the LV wedge model, a surrounding bath of width 100 μm was defined on all cut faces, with the width determined by the geometry of the MR-data bordering epi- and endocardial surfaces.

### 2.2. Simulation of electrical activity

Electrical activation within the ventricular model was simulated using a monodomain representation using the Cardiac Arrhythmia Research Package (CARP), the specifics of the numerical regimes of which have been described extensively elsewhere (Vigmond et al., 2003).

Experimentally-derived conductivities were assigned along the fiber and cross-fiber directions within the intracellular and extracellular domains (Clerc, 1976). Bath conductivity was set to 1.0 S/m, with vessel lumen wall conductivity 0.01 S/m. Cell membrane dynamics within the myocardial tissue were represented by the recent Mahajan-Shiferaw rabbit ventricular cell model (Mahajan et al., 2008).

Simulations of electrophysiological dynamics were performed as the first step in the pipeline, prior to optical mapping photon scattering simulation, thus providing *V*_*m*_ values at 1 ms (or less) discretization across all finite element nodes within the models. Two stimulation protocols were used in both simplistic and anatomically-detailed wedge models to initiate wavefront propagation circumferentially (approximately parallel to epicardial recording surface, following stimulation of a transmural cut face) and in a transmural direction (approximately toward epicardial recording surface, following stimulation of the endocardium).

Strong extracellular S2 shocks were also applied to the simplified cuboid models via plate electrodes in the *yz*-plane, located at the extremities of the extracellular bath in the *x*-direction. Shock waveforms were square, mono-phasic and of 5 ms duration. Shock strengths used were 10, 20, and 40 V, applied to diastolic tissue. Analysis was performed on *V*_*m*_ distributions at shock-end.

### 2.3. Basic photon transport simulation using MC

The fundamental algorithm used to simulate the step-by-step propagation and interaction of photons through cardiac tissue is based-on that of Wang et al. (1995). The algorithm describes the transport of photons through multi-layered biological tissue within a structured, regular domain, discretized into equal cubic optical elements into which physical quantities are stored.

#### 2.3.1. Photon propagation

Briefly, photons are propagated through the tissue in packets, each of which has an associated packet weight, *W*. At any time, the photon packet's position is described by Cartesian coordinates *x*, *y*, *z*. Its current direction of movement is defined by two angles, the deflection angle θ and the azimuthal angle ψ, from which the *directional cosines* μ_*x*_, μ_*y*_, and μ_*z*_ may be defined

(1) $$\mu_{x}=r\;\cdot \;\hat{x},\;\mu_{y}=r\;\cdot \;\hat{y},\;\mu_{z}=r\;\cdot \;\hat{z}$$

where **r** represents the current direction of propagation and $\hat{x}$, **ŷ**, and **ẑ** are Cartesian unit vectors.

The photon packets move in successive, free-fly steps in which neither absorption nor scattering occurs. The distance of each step size, *s*, depends upon the local optical properties, specifically the tissue absorption and scattering coefficients (μ_*a*_ and μ_*s*_, respectively). and involves sampling from a probability distribution.

(2) $$s=-\ln\;(\xi )/\mu_{t}$$

where ξ is a random uniformly distributed variable 0 ≤ ξ ≤ 1 and μ_*t*_ is the optical interaction coefficient, defined as μ_*t*_ = μ_*a*_ + μ_*s*_. The photon packet is then advanced forward by *s*, so long as interaction with a boundary does not occur, into its new position

(3) $$x^{\prime }=x+\mu_{x}s\;y^{\prime }=y+\mu_{y}s\;z^{\prime }=z+\mu_{z}s.$$

Once at its new location, the photon packet interacts with the tissue by firstly depositing a proportion of its weight, given by

(4) $$\delta W=W\mu_{a}/\left(\mu_{a}+\mu_{s}\right),$$

via absorption into the current optical element in which it resides. It is important to note that determining the optical element in which a photon packet will reside after moving to its new location (*x*′, *y*′, *z*′) is relatively trivial in regular geometries (cuboid/slab, etc.), through knowledge of the resolution of the optical grid. Such a method must be highly computationally-efficient as it must be evaluated numerous times for each individual photon packet trajectory for each of the many millions of packets launched.

Following absorption, the remaining packet weight is then scattered into a new direction which depends upon the current direction of travel and the optical anisotropy of scattering of the material, *g*, and is determined by the Henyey-Greenstein function (Wang et al., 1995). Note that a value of *g* = 0 represents isotropic scattering, whereas *g* = 1 or *g* = − 1 represents forward and back scattering, respectively. The scattering function also includes additional stochasticity from the use of additional random numbers. Photon propagation continues, absorbing and scattering between steps until the packet weight falls below a certain threshold, at which point it is either terminated and a new packet initiated, or else given the chance to continue propagating, in keeping with the conservation of energy (Wang et al., 1995).

#### 2.3.2. Interaction with boundaries

During its journey the photon packet may attempt to cross-over and interact with a boundary separating two regions with different optical properties. This boundary could, if propagating within an inhomogeneous medium, represent an internal boundary between different regions of the tissue, or else it could represent an external boundary at the edge of the tissue domain. When such an event occurs, the photon packet may be internally reflected or else transmitted through into the adjoining medium. Snell's Law is used to derive the angle of transmittance α_*t*_ from the known angle of incidence α_*i*_ and, along with Fresnel's formula, the internal reflectance *R* calculated to determine the probability of reflecting. If reflected or transmitted, the photon packet continues by moving the remaining distance from its initial step-size. For external boundaries, upon transmission the packet may be tracked through the surrounding media until it potentially passes back into the tissue medium or interacts with the detection device (Bishop et al., 2009), or alternatively it is killed and another packet launched.

In a regular, structured domain, keeping track of these interactions is relatively straight-forward as the definition of surfaces is usually simpler (and regular) with relatively few planar surface definitions within the model, allowing fast and efficient checks to be performed which do not hinder computational performance of the scattering algorithm. However, in a highly unstructured environment, such as that represented by the finite element LV wedge model of Figure 1B, this process is significantly more complex. Due to its unstructured nature, each triangle defining the interface between different media represent their own (often unique) planar surface. Thus, performing checks for intersection on such a large number of surfaces for each photon trajectory at each step becomes rapidly computationally intractable.

### 2.4. Photon transport simulation in unstructured tetrahedral finite element models

The main algorithm used to simulate the propagation and interaction of photons through the finite element meshes representing cardiac tissue during both the processes of illumination and fluorescent emission was based upon that of Shen and Wang (2010) which describes a tetrahedron-based inhomogeneous MC optical simulator, built upon that of Wang et al. (1995). To model the interaction of photons with, and transport through, the perfusing bath, in addition to other specific features of the optical mapping system, adaptations to this method were necessary, described below.

#### 2.4.1. Face-boundary interaction checking

The algorithm introduced by Shen and Wang (2010) introduces a fast and efficient procedure for determining photon-triangle interactions recursively and rapidly within an inhomogeneous medium defined by tetrahedral elements.

In the case of a domain defined by an unstructured tetrahedral finite element mesh, at any time, a photon packet resides within a tetrahedral element having four triangular faces. During each photon packet step, a rapid test can be performed to assess whether the photon trajectory intersects with one of these four boundaries.

**Figure 2:**
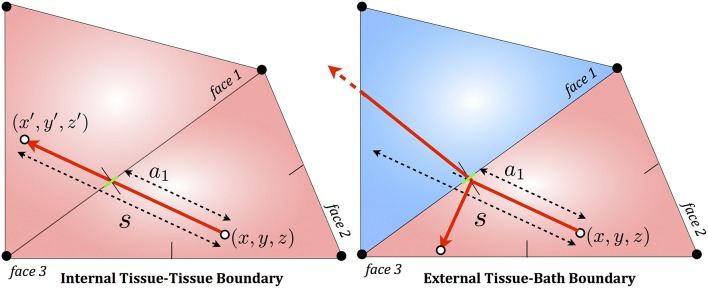
**2D schematic of triangular face boundary interaction algorithm for a photon packet attempting to propagate through a boundary between two tissue elements (left) or between a tissue element and a bath element (right)**. *s* represents the total step-size of the packet, attempting to move between its initial position at *x*, *y*, *z* to its final position at *x*′, *y*′, *z*′. Moving along this trajectory, it encounters a boundary face (face 1), at a distance *a*_1_ from *x*, *y*, *z*. Note that distances to other faces 2 and 3 (*a*_2_ and *a*_3_) are negative. The site of interaction is shown by a thin green line. In the case of two tissue elements (**left**), the packet's path is undisturbed and it continues to move its remaining step-size (*s* − *a*_1_) in the adjacent element into which it passes. In the case of the boundary between tissue and bath (**right**) the photon packet experiences either reflection back into the tissue element, or transmission into the bath, subject to refraction.

From each triangular boundary, an in-ward pointing surface-normal vector can be found, defined as pointing toward the centroid of the tetrahedron, shown in Figure 2. In our implementation, the distance of the starting position *x*, *y*, *z* to the plane along the direction of photon propagation (*a*_*j*_) is calculated for each triangular face *j*. If any *a*_*j*_ < *s* and *a*_*j*_ is positive, then interaction has occurred. If no interaction has occurred, the photon simply moves to its new position *x*′, *y*′, *z*′ which must also reside within the same tetrahedral as the packet has not exited the element.

If an interaction does occur, the packet will be moved to the first intersection point (lowest positive *a*_*j*_). Then, if the bordering tetrahedron which shares this triangular face boundary is part of the same medium, the packet continues to propagate into this new tetrahedron, checking again each of the four faces for possible intersections with the packet's remaining step-size of its trajectory, *s* − *a*_min_. If the neighboring element is part of a different medium, then reflection or transmission occurs in a similar manner to that described above for regular media. Such a process continues until the photon packet has moved its entire distance *s* during this particular step, with absorption and scattering occurring within the element that the photon packet resides at the end of this step. The procedure is then repeated for new steps until the photon packet is terminated, as described above.

The power of this method is that the element in which the packet resides is continually tracked as it propagates through the tissue, making it easy to assess when a packet attempts to cross-over a boundary between two different optical media. Furthermore, for each sub-step of the packet's main free-fly step (as it moves from element to element), only four intersection checks need be performed. Full details of the fundamental algorithm can be found in Shen and Wang (2010). A (2D) schematic of the boundary interaction method is shown in Figure 2.

#### 2.4.2. Element connectivity

The speed of the above algorithm can be significantly improved by utilizing a series look-up tables, as applied extensively in our implementation. The most important of these is a table detailing the element face-to-face connectivity, which has length number of elements and 4 columns (one for each face). For each tetrahedral face of each element, the element number of the neighboring tetrahedron which also directly shares this same face is listed, which gives the element into which the photon packet will move if it passes through this particular face of this element. If the element has a face which forms the exterior boundary to the entire domain, then there will be no neighboring element into which to pass and thus a flag is added acknowledging this. Such a look-up table allows to very quickly trace the trajectory of a photon packet as it passes from element-to-element through the domain.

An additional look-up table can also be constructed which specifically identifies external surface boundaries. Such a table has the same dimensions as the above element face-to-face connectivity, but instead specifies which faces represent boundaries between different optical domains, including exterior faces.

#### 2.4.3. Adaptation for photon movement within the bath

One important modification is required to the above algorithm in the case of simulating optical mapping signals. During optical mapping experiments, the cardiac tissue is continually perfused by saline solution. In some cases, the entire preparation sits submerged within an extensive bath of dimensions much larger than the preparation itself (Bishop et al., 2007, 2011b). In the simple first approximation, the domain of interest is composed of just two optical media types, myocardial tissue and saline solution (or bath). When photons exit the myocardial tissue they therefore enter the bath. Although the photons both scatter and absorb readily in the myocardial tissue, this is not the case in the bath. Here, the interaction rate is significantly lower with the photons traveling a significantly larger free-fly step-size before absorbing and scattering. In an optical mapping set-up, the distances that photons may travel within the bath medium is still relatively small compared to this large interaction distance. Therefore, in our specific case of simulating optical mapping signals we assume that the photons move freely throughout the bath medium, with straight-line trajectories neither undergoing absorption nor scattering. Thus, when a photon exits the myocardial tissue into the bath, its trajectory is traced in a straight-line until it either passes out of the domain entirely (as would usually be the case for photons exiting the epicardial surface) or alternatively intersects with myocardial tissue once again (as would happen in the case when it exits a cavity surface). In the latter case, the same boundary interaction method as above is followed with the packet either being transmitted into the myocardium at this point once again or being reflected back into the bath. During its propagation through the bath medium, the packet loses no weight (no absorption), its current step-size is not reduced and the remaining step-length and weight (which is had when it left) is continued once it re-enters the tissue.

### 2.5. Simulation of fluorescent optical mapping signals

The above photon transport algorithm is used to simulate both the processes of excitation illumination and (voltage-sensitive) fluorescent emission, using optical properties (μ_*a*_, μ_*s*_, *g*) obtained at the wavelengths specific to illumination and emission (Ding et al., 2001), which together form the foundations for the production of the signal recorded during cardiac optical mapping.

#### 2.5.1. Simulating illumination

To simulate uniform illumination of the tissue surface by an external source, packets are incident upon the external tissue boundary, at an illumination angle θ_illum_, usually taken at 0 to replicate illuminated light normal to the tissue surface (Ding et al., 2001; Bishop et al., 2009). As the photon packets propagate through the tissue they deposit weight within the tissue which is logged in the optical elements, which thus represents photon density (photons per volume) due to illumination throughout the tissue. Optical parameters of cardiac tissue measured at the typical illumination wavelength (488 nm) of the commonly-used voltage-sensitive dye DI-4-ANNEPS are μ_*a*_ = 0.52, μ_*s*_ = 23.0 and *g* = 0.94 (Ding et al., 2001).

#### 2.5.2. Simulating fluorescent emission

In the case of fluorescent emission, the fluorescent photons originate from dye molecules within the tissue itself; the more photons received by a region of tissue during illumination, the more fluorescent dye molecules get excited and fluoresce. To simulate this process, the total number of fluorescent photon packets emitted from each optical element within the tissue is directly proportional to the corresponding excitation photon density at that same element. In contrast to the process of illumination, fluorescent photon packets are emitted at randomly distributed angles (isotropic emission) from their point source within the tissue. Optical parameters of cardiac tissue measured at the typical emission wavelength (669 nm) of the commonly-used voltage-sensitive dye DI-4-ANNEPS are μ_*a*_ = 0.1, μ_*s*_ = 21.8, and *g* = 0.96 (Ding et al., 2001).

#### 2.5.3. Simulating optical detection

If a photon packet exits the tissue surface within an area from which signals are being recorded, it deposits its total weight at the time of exit as the recorded fluorescent signal from that particular region, or “pixel,” of imaged tissue. Successive photon packets exiting the same region build-up the total signal recorded from this pixel, with the total recorded intensity thus representing the final accumulated packet weight. Furthermore, each and every fluorescent photon packet which exits from this specific region on the tissue surface contains information regarding its point of origin within the tissue. With this information, along with the weight of the exiting packet, a distribution can be built-up which shows the relative fraction of recorded fluorescence that originates from a given volume of tissue, termed the scattering or interrogation volume (Ding et al., 2001; Bishop et al., 2006a, 2009), which thus provides essential information regarding the origin of optical mapping signals under different circumstances. In this study, photons exiting the tissue at all angles were recorded as “detected.”

#### 2.5.4. Simulating voltage-sensitive fluorescent emission

The computed scattering volumes for each detection site provides quantification of the relative fraction of recorded fluorescence that originates from different regions of tissue beneath the detection pixel. This information is then be convoluted with the calculated distribution of *V*_*m*_ at corresponding points throughout the tissue to simulate *voltage-sensitive* fluorescent emission (Bishop et al., 2009). *V*_opt_ represents the total signal intensity collected at a given optical detection site. Note that *V*_*m*_ can be normalized and scaled such that the total recorded fluorescence faithfully replicates the experimental scenario of an approximate 10% change upon a background of fluorescence. Distributions of *V*_*m*_ at sequential outputted time intervals (0.2 − 1.0 ms, for example) can thus be used to create a time-varying simulated optical signal.

### 2.6. Data analysis

The total accumulated weight of photon packets exiting pixels of differing square edge-lengths (160, 320, 640 μm) at chosen locations on the epicardial surface (Figure 1A) was recorded as the total optical signal, *V*_opt_. Action potential upstroke durations were defined as the time interval between 10 and 90% depolarization for simulated *V*_opt_ signals. *V*_opt_ action potentials were normalized between the resting and maximum depolarized values. In addition to total optical signals recorded from a pixel, *scattering volumes* were constructed for each pixel during fluorescent emission, detailing the relative contribution from each optical tissue element in the domain to the total signal recorded from that particular pixel. Here, normalized scattering volumes are plotted over the meshes whereby the relative contribution from each optical element is scaled to the maximal contribution value within the mesh.

Photon diffusion theory says that in tissue in which scattering dominates over absorption, the photon diffusion equation is valid (Arridge, 1993; Jacques, 1998). For uniform illumination over the surface of a semi-infinite plane or slab, diffusion theory gives the decay of photon density Φ into the tissue as

(5) $$\Phi (z)=\Phi_{0}\exp\;(-z/\delta ),$$

where *z* is the depth into the tissue and δ is the penetration depth, with a value given by

(6) $$\delta =\sqrt{\frac{D}{\mu_{a}}},$$

where *D* is the diffusion coefficient and is equal to *D* = $\frac{1}{3(\mu_{a}+\mu_{s}(1-g))}$. We note here that the analytic solution to the photon diffusion equation in the case of a slab of finite thickness is actually of a more complex form to that showed above (Bernus et al., 2005). Although in this study we use a slab of finite thickness, we refer to this solution for the more simple case of a semi-infinite slab as an approximation which may be expected to be good for relatively thick slabs (4 mm) with respect to the penetration depth of the illuminating light (δ_illum_ = 0.59 used here.)

## 3. Results

### 3.1. Illumination

**Figure 3:**
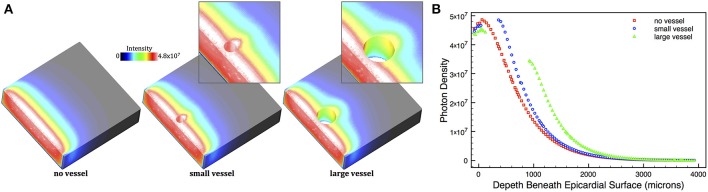
**(A)** Distribution of photon density within the simple cuboid models following uniform epicardial illumination, shown as cuts through central *xy*-plane. Regions around cavities in the vessel models are highlighted. **(B)** Depth profile of illumination photon density taken along the *x*-axis central to the *yz*-plane in the no vessel (red squares), small vessel (blue circles), and large vessel (green triangles) models.

Excitation illumination was simulated using the MC algorithm described above in Section 2.5.1. 50,000 photon packets were incident from every epicardial surface triangle in the simple cuboid models and the total accumulated photon weight deposited within the tissue logged. Figure 3A shows the total deposited photon density within the models. Here, we clearly see the attenuation of photon density with depth away from the illuminated epicardial surface in all models. However, in regions around the subepicardial vessels in the small and large vessel models the distribution of photon density is distorted, emphasized by the corresponding highlighted regions. Photon density around the distal side of the vessel cavity relative to the epicardium is higher compared to a similar depth in the no vessel model. This difference is emphasized in Figure 3B, which plots the profile of illumination photon density with depth beneath the surface for all models. For example, the photon density on the distal side of the cavity in the large vessel model is over twice as large (3.4 × 10^7^), compared to the corresponding depth location (1000 μm) in the no vessel model (1.6 × 10^7^). In the small vessel model, this difference is less significant, but still over 30% larger (4.9 × 10^7^ in the vessel model, compared to 3.7 × 10^7^ in the no vessel model at a depth of 450 μm).

The overall profiles of the illumination photon density with depth, shown in Figure 3, are fundamentally of a mono-exponential form, with a small subsurface peak, as expected (Hyatt et al., 2008; Bishop et al., 2009). Analysis of the profile of the no vessel model for this specific cuboid geometry demonstrated that it decayed marginally more rapidly than a simple exponential, due to photon escape from the surfaces at *z* = ±2 mm, reflecting the fact that the mono-exponential form of Equation 5 is only an approximation for the case of a finite thickness slab (Bernus et al., 2005).

**Figure 4:**
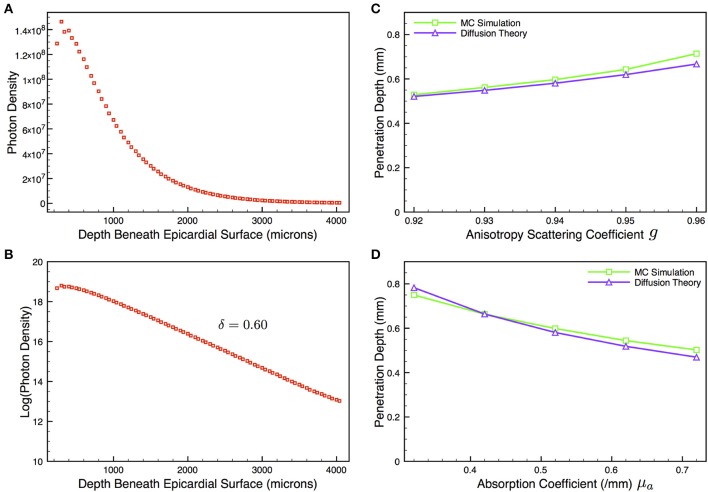
**Depth profile of illumination photon density (A) within thicker no vessel model of dimensions 4 × 4 × 4 mm taken along the *x*-axis central to the *yz*-plane, with corresponding log-plot (B) to confirm mono-exponential nature of decay in depth**. Gradient of linear region of log-plot (>1000 μm) gives penetration depth δ = 0.60 mm. **(C,D)** show variation in δ from MC simulations (green squares) and that predicted from diffusion theory (purple triangles) as optical absorptivity μ_*a*_ and scattering coefficient *g* are varied.

In order to validate our model to assess whether it behaved in approximately the expected manner upon variation of optical absorption and scattering parameters, as predicted from diffusion theory in the case of a semi-infinite slab, we used an additional solid (no vessel) model of larger dimensions (4 × 4 × 4 mm) and simulated illumination in a similar manner to above. Figure 4A shows the plot with depth of the illumination photon density in this case, with Figure 4B showing the corresponding log-plot. As can be seen from Figure 4B, the decay of photon density with depth within this thicker slab was very close to a mono-exponential for depths > 1000 μm, giving a penetration depth of δ = 0.60 mm, compared to the theoretical value of 0.58 mm predicted from the diffusion theory approximation in the case of a semi-infinite slab. Figures 4C,D now show the variation in the δ values derived from the approximate fitted mono-exponential for our MC model and that predicted from diffusion theory in the case of a semi-infinite slab (simply derived directly from Equation 5) as the optical parameters μ_*a*_ and *g* are varied to change the optical absorption and scattering properties of the tissue, respectively. Both plots demonstrate that our MC model compares well with the expected change in penetration depth δ as μ_*a*_ and *g* are varied as predicted from diffusion theory. Despite the mono-exponential solution to the diffusion equation only being valid in the case of semi-infinite slabs, the approximation in the case of the 4 mm cube used here is expected to be good, and thus the plotted variation of δ with μ_*a*_ and *g* provides a good indication that our model is behaving as expected upon parameter variation.

### 3.2. Fluorescent emission

**Figure 5:**
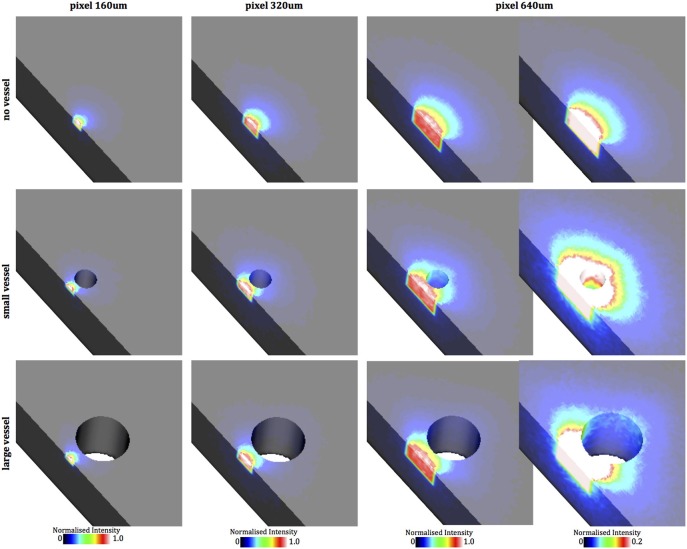
**Plots of the relative contribution of different spatial regions within the tissue to the total fluorescent signal recorded by a given pixel, normalized to the maximal contribution value, (the normalized scattering volume) for pixel sizes 160, 320, and 640 μm for each of the simple cuboid models, shown as cuts through central *xy*-plane**. In the case of the 640 μm pixel, an additional set of plots is shown with maximum of the color-bar scaled to 0.2% of the max intensity to highlight contributions from deeper tissue regions.

Following uniform epicardial illumination, fluorescent emission was simulated as described in Section 2.5.2. Figure 5 shows the normalized scattering volumes (defined in Section 2.6) for pixels of edge-lengths 160, 320, and 640 μm for each model. Firstly, Figure 5 shows that large pixel dimensions collect more photons from a more largely distributed spatial region beneath the recording surface. More importantly, though, the presence of the cavity in the small and large vessel models significantly distorts the dimensions of the scattering volume, making it extend deeper into the tissue relative to the case of the no vessel model, more apparent as the pixel size increases and for the larger vessel. This effect is most noticeable in the right-hand panel of the 640 μm pixel where the maximum color-bar scale has been adjusted to 20% of the max intensity to more clearly highlight contributions from deeper tissue regions.

**Figure 6:**
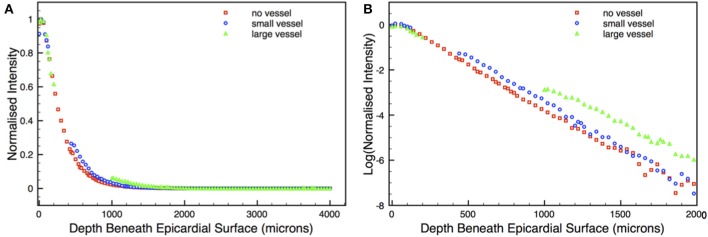
**Depth profile of the normalized scattering volume intensity for the 640 μm pixel along the *x*-axis central to the *yz*-plane (A) with corresponding log-plot (B) for no vessel (red squares), small vessel (blue circles), and large vessel (green triangles) models**.

The deeper penetration of the scattering volumes seen in the vessel models is further highlighted in Figure 6A which shows depth profiles of the normalized scattering volume contributions for the 640 μm pixel, with Figure 6B showing a corresponding log-plot to emphasize differences. Although the contributions to the total recorded signal are relatively minor in these regions, the value at the cavity edge distal to the epicardium in the large vessel model is still almost 3-fold larger than the corresponding location (1000 μm depth) in the no vessel model (0.059 vs. 0.022 of the normalized scattering volume intensity). In the small vessel model it is 0.281 (at 450 μm depth) compared to 0.215 in the no vessel model.

### 3.3. Voltage-sensitive fluorescent emission

**Figure 7:**
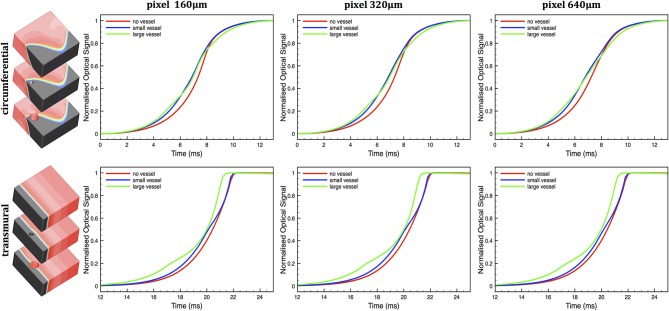
**Simulated optical *V*_opt_ action potential upstrokes during circumferential (top) and transmural (bottom) wavefront propagation in the no vessel (red), small vessel (blue) and large vessel (green) cuboid models for pixel sizes 160, 320, and 640 μm**.

The functional consequence of these differences in scattering volumes from surface locations near to cavities was assessed by using them to simulate voltage-sensitive fluorescent signals, as described in Section 2.5.4. Voltage-sensitive fluorescent signals were simulated during wavefront propagation through the simple cuboid models in both circumferential and transmural propagation directions, as described in Section 2.2. Figure 7 shows simulated optical signal *V*_opt_ upstrokes in the simple cuboid models for both circumferential (top) and transmural (bottom) electrical propagation, for each pixel size.

In the case of circumferential propagation, the action potential upstroke morphologies are largely similar between different models, all showing the well-known prolongation with respect to the raw electrical *V*_*m*_ upstroke (≈ 1 − 2 ms), whilst remaining approximately symmetrical. However, the vessel models show a more significantly prolonged upstroke for all pixel sizes, with the large vessel model upstrokes longer than the small vessel upstrokes; for example upstrokes of 5.70 ms (large vessel), 5.11 ms (small vessel) and 4.74 ms (no vessel) are witnessed for the 640 μm pixel. Note that little difference was seen between upstroke duration for different pixel sizes.

In the case of transmural propagation, more significant differences are witnessed in upstroke morphology between the models. All models show the well-known asymmetrical prolongation for wavefront propagation toward the recording site, with the lower part of the upstroke being prolonged, with the upper part less so (Hyatt et al., 2003, 2005; Bishop et al., 2006a). However, the vessel models, particularly the large vessel model, show a significant “hump” in the upstroke profile at low polarization levels (0.1 − 0.4) for all pixel sizes. In addition, the vessel models again show a greater overall upstroke prolongation than the no vessel model, being, for example. 5.08 ms (large vessel), 4.55 ms (small vessel) and 4.24 ms (no vessel) for the 640 μm pixel.

**Figure 8:**
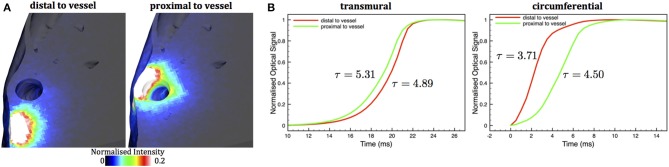
**(A)** Normalized scattering volumes associated with 640 μm pixels close to (right) and distant from (left) a large subepicardial vessel cavity in the anatomically-detailed wedge model. Maximum of the color-bar is scaled to 0.2 of the maximum intensity to highlight contributions from deeper tissue regions. **(B)** Simulated optical *V*_opt_ action potential upstrokes during transmural (left) and circumferential (right) wavefront propagation in the wedge model corresponding to pixel locations close to (green) or distant from (red) a large subepicardial vessel.

Finally, we demonstrate the applicability of our method to simulate fluorescent voltage-sensitive signals from the anatomically-detailed wedge model. Figure 8A shows the spatial distributions of two different scattering volumes corresponding to pixels (edge-length 640 μm) on the epicardial surface of the wedge model both close to (right) and distant (left) to a large subepicardial vessel. Similarly to the case of the simple models in Figure 5, the scattering volume associated with the pixel above the large subepicardial vessel is distorted with respect to the volume above the compact tissue, extending more deeply into the myocardium. Figure 8B shows the *V*_opt_ action potential upstrokes following simulation of voltage-sensitive fluorescent emission using these scattering volumes along with spatial distributions of *V*_*m*_ following circumferential and transmural pacing. Again, similarly to the simple models in Figure 7, the upstrokes recorded close to the large vessel is prolonged to a greater degree than that recorded away from the vessel: 4.50/5.31 ms close to the vessel and 3.71/4.50 ms distant to the vessel for circumferential and transmural pacing, respectively. Furthermore, the distortion present in the upstroke morphology following transmural pacing is, although apparent, less evident than in the simplified cuboid models. Note that, as upstrokes are recorded from slightly different spatial locations, slight differences in overall activation times are also apparent. In the case of circumferential pacing, the foot of the action potential is also slightly less prolonged than the corresponding no vessel case in Figure 7 due to its close proximity to the pacing site, which also leads to the wavefront being less curved as it passes under the recording pixel.

### 3.4. Simulation of shock-end virtual-electrode measurements

**Figure 9:**
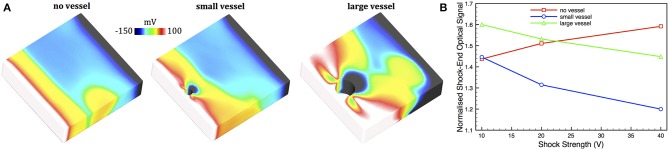
**(A)** *V*_*m*_ shock-end distributions in each of the simple cuboid models for a shock strength of 20 V, where a clipping plane along the central *xy* plane has been used to expose intramural tissue. **(B)** Simulated optical shock-end signals from a 320 μm pixel for shock-strengths of 10, 20, and 40 V for each of the no vessel (red squares), small vessel (blue circles), and large vessel (green triangles) models. Optical signals are normalized with respect to the action potential amplitude following pacing.

*V*_*m*_ distributions at the end of extra-cellular shocks applied to simple cuboid models (as described in Section 2.2) were used along with the scattering volumes shown in Figure 5 to compute simulated optical shock-end signals. Figure 9A shows the intramural *V*_*m*_ shock-end distributions in each of the models for a shock strength of 20 V. As expected, following the shock, the electrophysiological simulation shows strong *V*_*m*_ polarization levels on the surfaces, with the epicardial surface (closest to the cathode) strongly depolarized (>100 mV) and the endocardial surface (nearest the anode) strongly hyperpolarized (< − 150 mV). However, for strong shock-strengths, there exists a complex distribution of polarization levels around the vessel cavities, with the side of the vessel cavity distal to the epicardium becoming strongly hyperpolarized and the side proximal to the epicardium depolarized.

Figure 9B shows the corresponding simulated optical signals for shock-strengths between 10 and 40 V for each of the models corresponding to a pixel of edge-length 320 μm. The Figure shows that the optical signal predicted by the no vessel model increases, as expected, with applied shock-strength, experiencing polarization levels of 143% action potential amplitude at 10 V, and reaching 160% at 40 V.

For weak shocks, the small vessel model predicts similar shock-end polarization levels to the no vessel model (143% action potential amplitude), with the large vessel model predicting larger polarizations of 160%. However, intriguingly. in contrast to the no vessel model, the signals recorded from surface locations above vessels show the opposite trend, decreasing in magnitude as shock-strength increases. At a shock-strength of 40 V, the large vessel model has decreased to a polarization level of 145% action potential amplitude, whereas the small vessel model has decreased further to just 120%.

## 4. Discussion

### 4.1. Utility of modeling methodology

We have introduced a novel method for the simulation of voltage-sensitive cardiac optical mapping signals using an adapted MC model of light transport. Our method presented here overcomes the previous limitations of continuum diffusion-based models, allowing photon propagation to be simulated through scattering media (tissue) and non-scattering media (saline-filled cavities) alike. Importantly, the model can therefore be applied to simulate fluorescent signals from the latest high-resolution anatomically-detailed computational geometries, including the presence of fine-scaled anatomical complexity such as intramural blood vessel cavities and extracellular cleft spaces (Bishop et al., 2010b), which was not possible using diffusion-based models. This has allowed us to investigate the intricate interaction of fluorescent signal distortion due to light penetration and photon scattering in the vicinity of such non-scattering intramural cavities, such as vessels, which has key relevance to combined modeling and experimental optical mapping studies. Firstly, it will provide an essential tool to facilitate a closer, and essential, validation of the predictions made from computational simulation results using these latest highly-detailed MR-based models with optical mapping recordings which, until now, has not been possible. Secondly, it will provide important insight into the underlying mechanisms of fluorescent signal distortion and the role of fine-scale structures, which may be of significant importance in facilitating a better interpretation of optical mapping signals from high-resolution imaging systems (Bub et al., 2010; Kelly et al., 2013).

### 4.2. Importance of light interaction with fine-scale features

Using our model, we have demonstrated the differences in fluorescent signals recorded close to subepicardial cavities and those from above regions of compact myocardium, which can, in certain circumstances, be significant. These differences (shown here as differences in optical scattering volumes, action potential upstrokes and shock-end polarization recordings) may, to a degree, explain pixel-by-pixl heterogeneity in optically-recorded electrophysiological metrics such as action potential upstrokes and durations, frequently seen in experimental recordings. Furthermore, the models used here have been derived-from, or base-on, rabbit MR-data. However, the relative degree of signal distortion due to intramural structures from larger species (such as pig, canine, or even human samples frequently used in optical mapping experiments) may be even more significant, due to the relatively larger sizes of the cavities and anatomy involved, whilst having similar optical properties for absorption and scattering. Such distortion effects could also have even more relevance in the use of optical dyes with longer excitation and/or emission wavelengths in which cardiac tissue is less absorbing and where signals are known to be collected from much larger scattering volumes (Walton et al., 2010).

We note here that extracellular cleft spaces we not represented in our simplified slab models, but were only present in the detailed MR-derived wedge model, and consequently a detailed analysis of their specific effects was not performed. However, although we believe that large extracellular cleft spaces (containing large regions of non-scattering media) have the potential to affect the optical signal in a similar manner to the vessel cavities investigated in detail in this study, the large extracellular clefts witnessed in the MR data tended not to be located near the epicardial surface, but were found more intramurally within the tissue. Thus, we suggest that extracellular cleft spaces play a lesser role in optical signal distortion.

### 4.3. Interpretation of upstroke measurements

The overall prolongation of the simulated optical action potential upstroke was similar to that reported experimentally (Girouard et al., 1996; Gray, 1999; Hyatt et al., 2005) and in other simulation studies using both diffusion and MC methods (Hyatt et al., 2003, 2005, 2008; Bishop et al., 2006a, 2009). In addition, the well-acknowledged difference in optical upstroke morphology for wavefronts propagating toward (transmural propagation) compared to parallel to (circumferential propagation) was also observed (Hyatt et al., 2003, 2005; Bishop et al., 2006a). However, our model allowed us to demonstrate important differences in both overall upstroke duration and upstroke morphology when recording signals in the viscinity of subsurface cavities, with upstroke duration being increased and having a noticeable “hump” at low polarization levels (transmural propagation), relative to recordings above compact tissue. Such differences may be explained by the important differences in scattering volumes highlighted in Figures 5, 6, 8, demonstrating how signals collected from above subsurface cavities contain a higher proportion of their intensity from deeper intramural depths, beneath the cavity itself. In the case of transmural propagation, this leads to earlier detection of the wavefront, causing the early “hump” in the upstroke. In the case of circumferential propagation, the nature of the intramural fiber architecture causes the wavefront to be curved (concaved), with intramural layers leading epi-/endocardial surface regions (Figure 7). Thus, collection of relatively more signal from intramural depths above a cavity leads to an earlier detection of the wavefront in these regions and thus a more prolonged upstroke duration.

Many optical mapping studies in recent years have used careful measurements of both upstroke durations and morphologies from the epicardial surface to infer detailed information regarding localized subsurface wavefront direction (Hyatt et al., 2003, 2005) and relative electrotonic loading (Kelly et al., 2013). Although, in this study we highlighted the significantly different effects on the upstroke due to the interaction of the wavefront with large sub-epicardial cavities for different overall *global* wavefront propagation directions (toward and parallel to the recording surface), the interaction with *localized subsurface* wavefront orientations with cavities may also represent an important consideration. The findings from this study therefore suggest careful interpretation of such optical recordings, in light of the exact surface location from which they are taken with respect to subsurface intramural cardiac anatomy.

### 4.4. Interpretation of shock-end fluorescent measurements

The collection of a significant fraction of the total fluorescent signal from a subsurface scattering volume of tissue beneath the surface recording site has previously been suggested to underlie the apparent reduction in optically-recorded “surface” fluorescent signals following strong extracellular shocks, relative to the magnitudes predicted by computational bidomain simulations (Janks and Roth, 2002; Bishop et al., 2006b, 2007). Such electrophysiological simulations show that polarization levels decrease rapidly into the tissue depth over length-scales of the order of a length constant, with intramural tissue correspondingly being of significantly weaker polarization levels than the strongly polarized external tissue surfaces (as shown in Figure 9A). Collecting signals from within the scattering volume has an averaging effect that modulates the recorded optical signal due to the inclusion of the more weakly-polarized intramural tissue. When combined with optical mapping signal synthesis models, simulations show a decrease in epicardial shock-end values, significantly reducing the disparity between simulations and experiments; however, the simulated values still consistently over-estimated those obtained experimentally (Bishop et al., 2007). The reduction in shock-end optical polarization levels seen in recordings above intramural vessel cavities undercovered in this study, relative to the stronger polarizations predicted above compact myocardial tissue, may go some way to explaining this previous disparity.

Recently, detailed modeling (Bishop et al., 2010a, 2012; Luther et al., 2012) [and experiments (Fast et al., 1998; Fast, 2002)] has also shown that intramural cavities can induce the formation of “virtual-electrodes” during strong extracellular shocks with opposite sides of the cavities becoming de-/hyper-polarized due to a movement of current out of, and back into, the intracellular domain as it traverses the cavity. The findings from our study (Figure 9) suggest that, as the complex distribution of polarization levels surrounding the cavities of subsurface vessels lies within the scattering volume, they make a significant contribution to the surface recorded optical signal.

Here, the reduced conductivity of the vessel lumen wall (Bishop et al., 2010a) leads to the epicardial side of the cavity becoming strongly depolarized with the distal mid-myocardial side becoming strongly hyperpolarized (Bishop et al., 2012). As shock strength increases, the virtual-electrode pattern around the vessel cavities becomes stronger and more wide-spread, more so for the larger vessel. This is more noticeable for the hyperpolarized tissue on the distal mid-myocardial side of the cavity, as there is less tissue on the epicardial side of the vessel which is already strongly depolarized by the shock anyway and so the amount of depolarized tissue in this region saturates. Due to the fact that the scattering volume extends beyond the distal side of the cavity (seen in Figure 5), these strongly hyperpolarized regions contribute to the total collected optical signal, reducing its magnitude with respect to recordings above compact myocardium where the scattering volume only samples from tissue which is either depolarized or of mid-polarization levels. As the subepicardial tissue is strongly depolarized by the shock anyway, the additional affect of the virtual-electrode from the vessel in this region does not have a major impact on the total recorded fluorescent signal (although it does appear to slightly increase the polarization level for the large vessel, evident at the weaker shock strengths). Thus, for stronger shocks, a larger amount of more hyperpolarized tissue on the distal side of the cavity contributes to the signal, causing optical polarization levels above the cavity to decrease with shock strength. The lack of this cavity-driven effect in the absence of vessels, causes the expected increase in simulated polarization levels with shock strength.

### 4.5. Study limitations

A potential limitation of our study is the relatively thin nature of the slab, particularly in the *z*- direction, and the resulting effect photon interactions with boundaries may have on the results presented here, which would not be expected to be present in the whole ventricular *in vivo* case. We have performed additional simulations using the larger no vessel model used in Section 3.1 and in Figure 4 of twice the thickness in the *z*-direction, being of dimension 4 × 4 × 4 mm, to investigate potential changes in the scattering volume due to boundary interactions. Our simulations showed no discernible differences in the spatial scattering volume within the model. More precisely, at the quoted depths of 450 and 1000 μm in Section 3.3, the values of the normalized intensity contributions to the scattering volume were 0.219 and 0.026, respectively, compared to the quoted values for the standard thinner no vessel model of 0.215 and 0.022, respectively. We therefore believe that the relatively small size of our models and the relatively close proximity of boundaries has not unduly affected the major findings of this study.

## 5. Conclusions

This study has presented a novel application of a MC photon scattering model to simulate, for the first time, cardiac optical mapping signals over anatomically-complex, unstructured, tetrahedral, finite element computational models, including representations of fine-scale anatomy and intramural cavities. This novel approach was used to demonstrate significant differences in optical action potential upstrokes (durations and morphologies) recorded above subepicardial vessels compared to those recorded above compact myocardial tissue, due to differences in subsurface optical signal collection volumes. Such differences were also responsible for significant reductions in the apparent optically-measured epicardial polarization above vessel cavities, compared to from tissue away from cavities. We have therefore demonstrated the importance of this novel optical mapping simulation approach along with highly anatomically-detailed models to fully investigate electrophysiological phenomena driven by fine-scale structural heterogeneity and to understand its recording by experimental imaging techniques.

### Conflict of interest statement

The Reviewer, Olivier Bernus, declares that despite having collaborated with the author Gernot Plank, the review process was handled objectively and no conflict of interest exists. The authors declare that the research was conducted in the absence of any commercial or financial relationships that could be construed as a potential conflict of interest.
